# Comprehensive analysis of cuproptosis-related lncRNAs model in tumor immune microenvironment and prognostic value of cervical cancer

**DOI:** 10.3389/fphar.2022.1065701

**Published:** 2022-11-30

**Authors:** Qiang Wang, Yue Xu

**Affiliations:** Department of Obstetrics and Gynecology, The Second Hospital of Jilin University, Changchun, China

**Keywords:** cervical cancer, cuproptosis, lncRNA, prognostic, antitumor drugs, tumor-infiltrating immune cells

## Abstract

Cervical cancer (CC) is the fourth leading gynecological malignancy in females worldwide. Cuproptosis, a form of cell death induced by copper, elicits a novel therapeutic strategy in anticancer therapy. Nonetheless, the effects of cuproptosis-related lncRNAs in CC remain unclear. Therefore, we aim to investigate cuproptosis-related lncRNAs, develop a risk model for prognostic prediction, and elucidate the immunological profile of CC. Transcription profiles and clinical follow-up data of CC were retrieved from The Cancer Genome Atlas (TCGA) database. Afterward, the risk model was built by distinguishing prognostic cuproptosis-related lncRNAs using the least absolute shrinkage and selection operator (LASSO) Cox regression. The correctness of the risk model was validated, and a nomogram was established followed by tumor immune microenvironment analysis. Tumor immune dysfunction and exclusion (TIDE) scores were used to assess immunotherapy response, and anticancer pharmaceutical half-maximal inhibitory concentration (IC50) prediction was performed for potential chemotherapy medicines. Finally, through coexpression analysis, 199 cuproptosis-related lncRNAs were collected. A unique risk model was generated using 6 selected prognostic cuproptosis-related lncRNAs. The risk score performed a reliable independent prediction of CC survival with higher diagnostic effectiveness compared to generic clinical characteristics. Immunological cell infiltration investigation indicated that the risk model was substantially linked with CC patients’ immunology, and the low-risk patients had lower TIDE scores and increased checkpoint expression, suggesting a stronger immunotherapy response. Besides, the high-risk group exhibited distinct sensitivity to anticancer medications. The immune-related progression was connected to the differentially expressed genes (DEGs) between risk groups. Generally, the risk model comprised 6 cuproptosis-related lncRNAs that may help predict CC patients’ overall survival, indicate immunocyte infiltration, and identify individualized treatment.

## Introduction

Cervical cancer (CC) is the fourth worst gynecological malignancy (Bhatla et al., 2021; Volkova et al., 2021). Annually, 604,000 new instances of cervical cancer are diagnosed, with 342,000 deaths. Persistent infection with high-risk human papillomavirus (HR-HPV) is essential for the pathogenesis of CC (Bhatla and Singhal, 2020). Despite immunization and early detection by cytological screening has significantly reduced the incidence rate, CC is still a substantial healthcare issue, particularly in low-to middle-income nations (Tsikouras et al., 2016; Sung et al., 2021). Additionally, molecular mechanisms underlying CC still have not been fully clarified and because of resistance and recurrence, the long-term prognosis for CC remains dismal (Small et al., 2017; Bhatla and Singhal, 2020). Therefore, it is imperative to identify accurate and reliable risk models for the prognostic prediction and personalized treatment strategy of CC.

Recently, research on cuprotosis in tumors has increased rapidly. Cuprotosis is a novel mechanism of nonapoptotic cell death that is strongly linked to mitochondrial respiration (Wang et al., 2022; Zhao et al., 2022). Cuproptosis has been involved in the pathogenesis of multiple tumors, including hepatocellular carcinoma (Zhang et al., 2022), gastric cancer (Feng et al., 2022), colon adenocarcinoma (Xu et al., 2022a), etc. The accumulation of copper in the cell results in cell dysfunction and death (Tsvetkov et al., 2022). These findings prompt the role of cuproptosis in tumor pathology with a promising therapeutic target for refractory cancers. However, research on the impacts and processes of cuproptosis in CC is still in its initial stages.

Long noncoding RNAs (lncRNAs), with transcripts > 200 nucleotides, have gained extensive interest recently as a potentially essential element of biological regulation. Growing evidence reveals that lncRNAs are commonly dysregulated in cervical malignancies. LncRNA PTENP1 induces cell apoptosis and inhibits CC cell proliferation (Fan et al., 2020). LncRNA SNHG1 enhances cell proliferation, migration, and invasion in CC (Liu et al., 2018). LINC01305 promotes the progression of CC (Huang et al., 2021). Besides, several lncRNAs, including ferroptosis-related (Jiang et al., 2022), N6-methyladenosine (m6A)-related (Zhang et al., 2022), and immune-related (Zheng et al., 2020) lncRNA signatures, have recently been implicated in CC patients’ pathophysiology and prognosis. However, the impact of lncRNAs in mediating cuproptosis in CC has not been addressed.

Here, we identified a unique 6-cuproptosis-related lncRNA risk model in CC patients for predicting the prognosis and chemotherapy responsiveness while also characterizing the tumor immune microenvironment. In line with expectations, our model showed preferred effectiveness in terms of overall survival (OS), and immune cell infiltration in CC patients as well as immunological checkpoints, and medication sensitivity, which might provide new insights into the personalized treatment of CC patients.

## Materials and methods

### Data procession

RNA-seq data of the TCGA-CESC cohort (309 CC samples, 3 noncancerous samples) was extracted from the TCGA database (https://tcga-data.nci.nih.gov/tcga/, 9 July 2022). Clinical data were retrieved. The lncRNA and mRNA sequence profiles were separated and compiled into a matrix respectively using the Perl programming language.

### Cuproptosis-associated lncRNAs collection

19 cuproptosis-related genes were retrieved from previous publications (Yang et al., 2022). Pearson’s correlational analysis was applied. Then, lncRNAs associated with cuproptosis were recognized (|R| > 0.4, *p* < 0.001) with the “limma” R package.

### Prognostic cuproptosis-related lncRNA risk model construction

Survival-associated cuproptosis-related lncRNAs were filtered using univariate Cox regression. Then, the risk lncRNAs were sorted out by LASSO with the “glmnet” R package. The penalty parameter (λ) was selected with minimum requirements. The multivariate Cox regression coefficient (β) was calculated. Each sample’s risk score was determined following the equation: risk score = ∑ (β_i_ × gene_i_ EXP) (EXP: expression level).

### Survival analysis

Based on median risk scores, patients were randomly assigned to low- or high-risk groups (ratio 1:1). The overall survival (OS) duration was evaluated in the two risk groups, as well as subgroups of different clinical features, utilizing the Kaplan-Meier (KM) survival analysis method with the “survminer” package.

### Nomogram establishment

The nomogram was established utilizing risk score, age, Grade, and T stage. The “rms” package was used to generate line graphs for the 1-, 3-, and 5-year OS with correction curves. The predictive capacity was determined using the Concordance Index (C-index) and time-dependent receiver operating characteristic (ROC) curve analysis with “survcomp” and “survivalROC” packages.

### Assessment of the risk score’s independent prognosis

The clinical characteristics of CC patients including Grade, T stage, age, and survival status, were examined in combination with the risk score by univariate and multivariate Cox regression analysis.

### Tumor immune analysis

We used the CIBERSORT method and the leukocyte gene signature matrix (LM22) as a reference to measure the immunocytes ratio in the tumor microenvironment (TME). We estimated the connection between risk scores and infiltrating immunocytes by comparing infiltrating immunocytes in the two risk groups with Wilcoxon signed-rank test. In addition, immune checkpoint gene expression was defined.

### Tumor immune dysfunction and exclusion assessment

The Tumor Immune Dysfunction and Exclusion (TIDE) approach can assess T cell malfunction, exclusion, and checkpoint inhibitors’ response (http://tide.dfci.harvard.edu/) (Jiang et al., 2018). Higher TIDE scores indicate a greater likelihood of antitumor immune escape.

### Functional enrichment analysis

DEGs of the two risk groups were identified (adjusted *p*-value < 0.05, |log_2_FC| > 1). Next, functional enrichment using Gene Ontology (GO), Kyoto Encyclopedia of Genes and Genomes (KEGG) pathway, and Disease Ontology (DO) analysis with “clusterProfiler” and “DOSE” packages was employed. We examined the alteration in gene sets between different risk cohorts with the MSigDB enrichment dataset using “GSEA” package (c2.cp.kegg.v7.4.symbols.gmt) with *p* value < 0.05 and *q* value < 0.05.

### Prediction of drug susceptibility

With the “pRRophetic” package (Geeleher et al., 2014), the half-maximal inhibitory concentrations (IC50) of anti-cancer medicines were administered to a variety of subgroups of patients to assess how effectively a substance inhibits a biological process. *p* < 0.001 was selected as the threshold.

### Statistical analysis

R (version 4.0.3) was used for statistical analysis. The Pearson chi-square test was applied for categorical data. Spearman’s correlation analysis was conducted to assess the correlation between risk score and drug sensitivity. A *p* < 0.05 was deemed significant.

## Results

### Identification of cuprotosis-related lncRNAs

**FIGURE 1:**
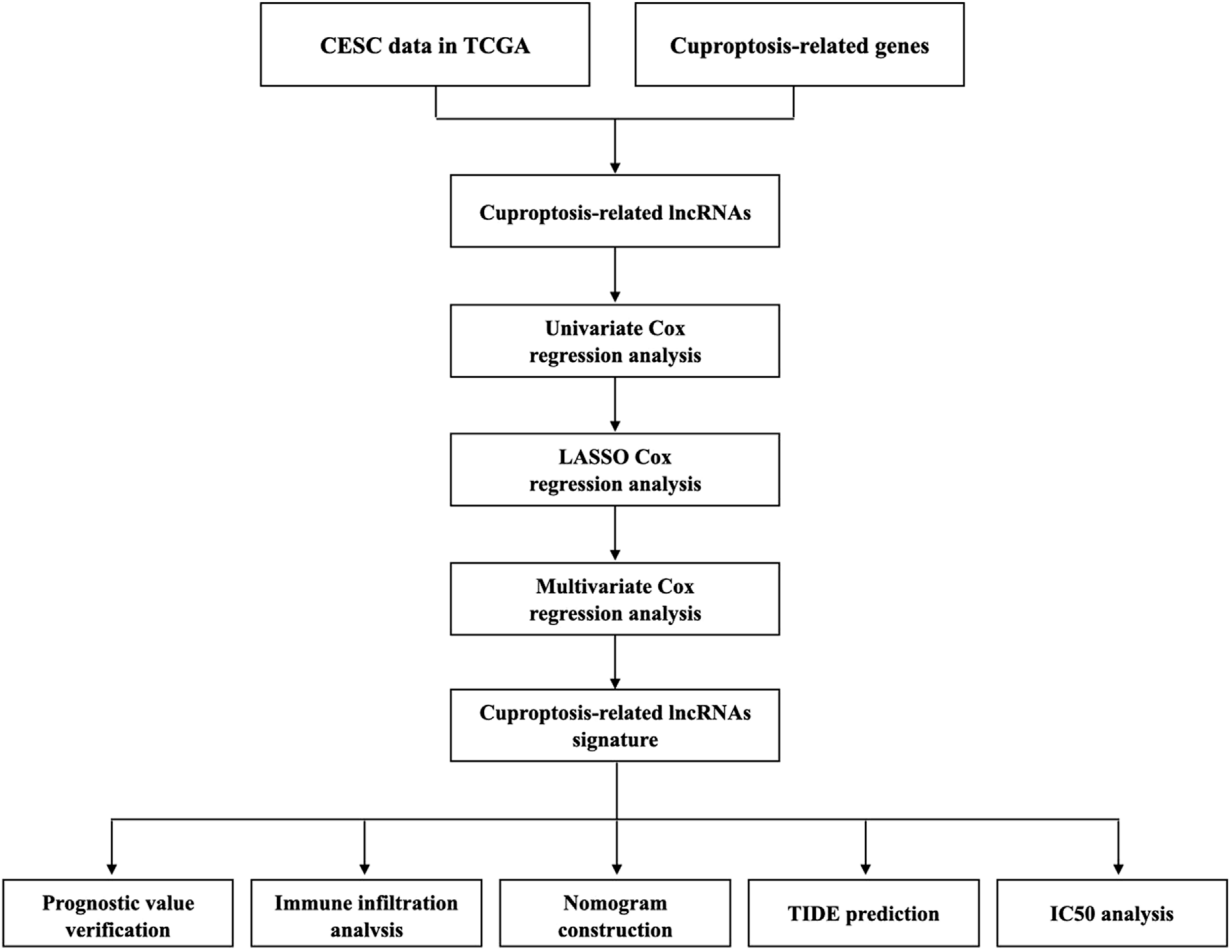
Workflow of the study.

**FIGURE 2:**
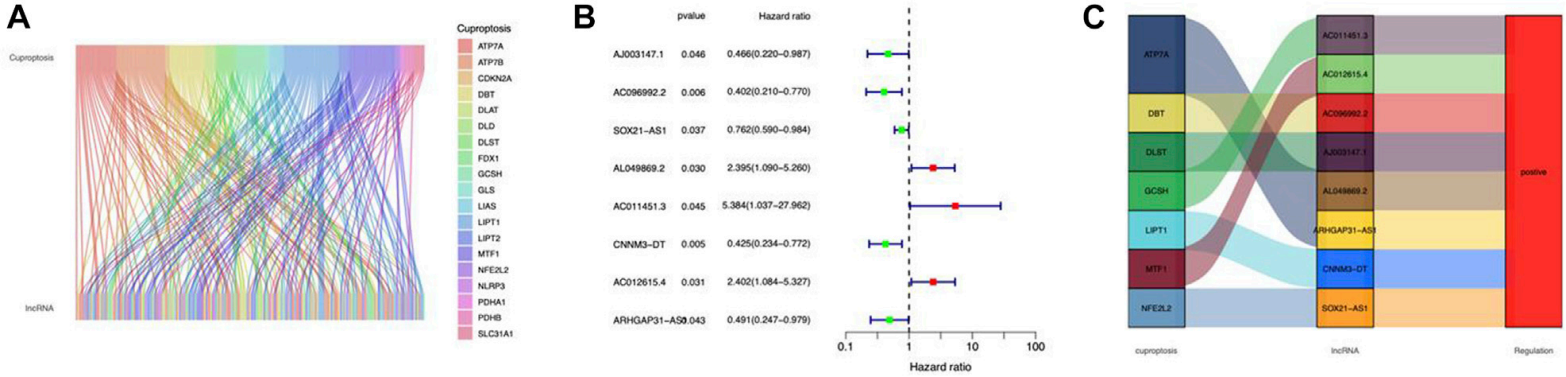
Identification of cuproptosis-related lncRNAs in CC. **(A)** Sankey relationship diagram of cuproptosis genes and cuproptosis-related lncRNAs. **(B)** The cuproptosis-related prognostic lncRNAs extracted by univariate Cox regression analysis. **(C)** The regulatory between cuproptosis-related genes and lncRNAs.

Figure 1 depicts our flow-process diagram. The cuproptosis-related genes and associated lncRNAs were visualized (Figure 2A). In total, 199 cuproptosis-related lncRNAs were filtered out. Of them, 91 were upregulated, and 89 were downregulated (adj. *P* < 0.05, |log_2_FC| > 1) (Supplementary Figures S1A,B). Univariate Cox regression analysis identified 8 prognostic cuproptosis-related lncRNAs (*p* < 0.05), of which, AJ003147.1, AC096992.2, SOX21-AS1, CNNM3-DT, and ARHGAP31-AS1 were protective factors for OS, and AL049869.2, AC011451.3, and AC012615.4 were risk factors for OS (Figure 2B). The 8 lncRNAs and cuproptosis genes showed a positive association (Figure 2C).

### Establishment of the risk model

**FIGURE 3:**
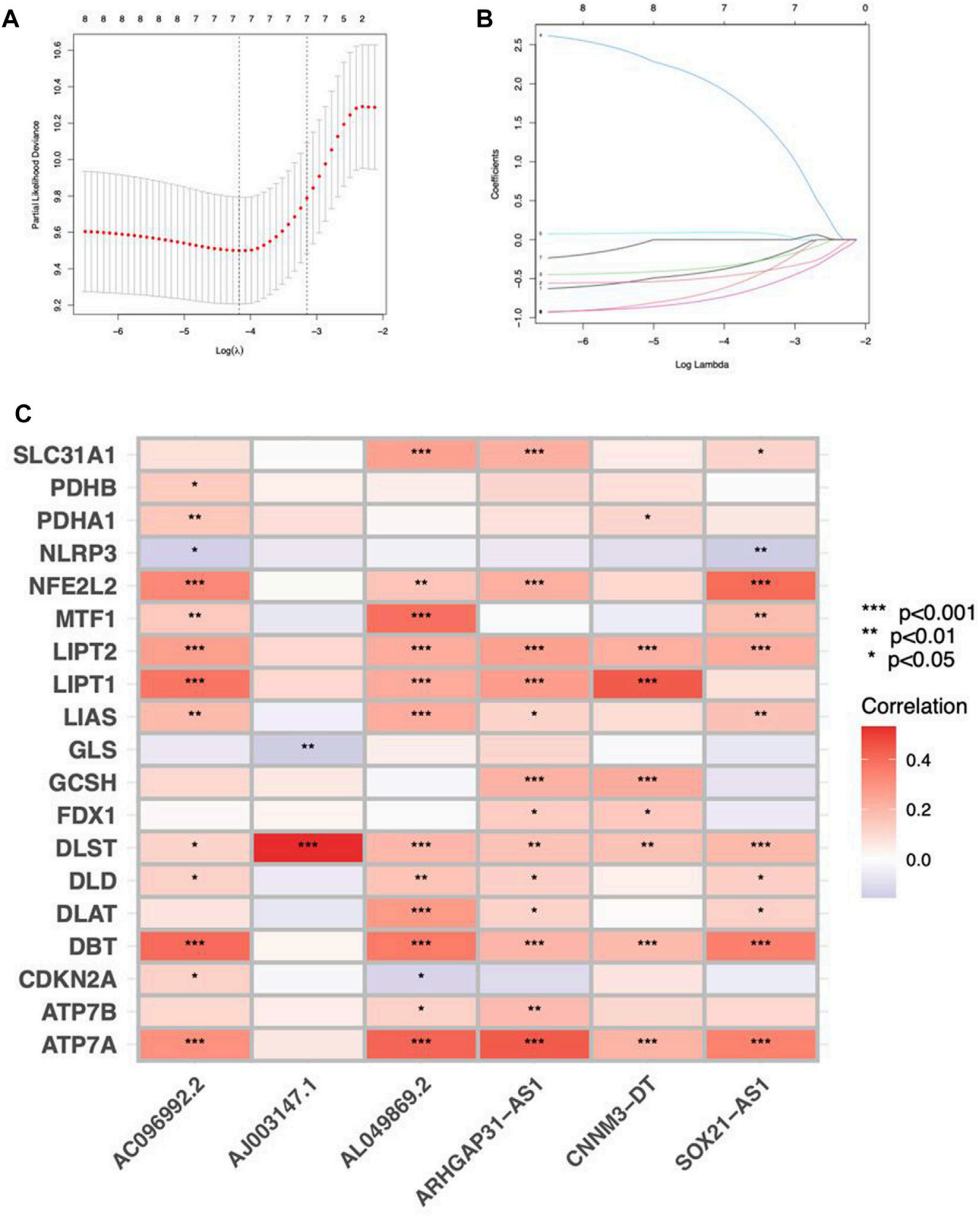
Construction of a prognostic risk model in CC. **(A)** A cross-validation procedure for optimizing LASSO regression parameters. **(B)** LASSO coefficient distribution. **(C)** The correlations between cuproptosis-associated genes and the 6 lncRNAs of the risk model.

**FIGURE 4:**
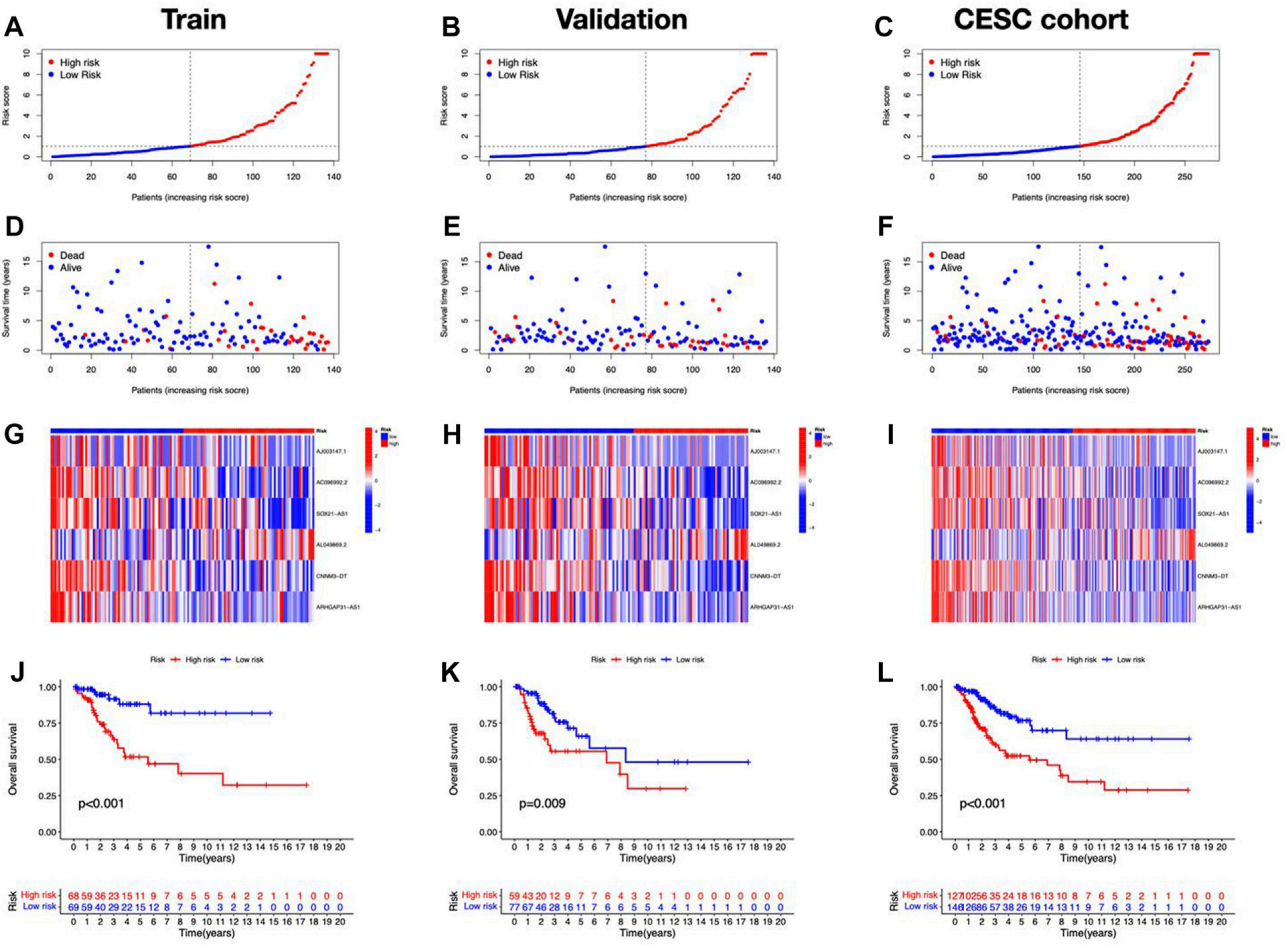
Prognostic performance of the risk model in TCGA dataset. The distribution of overall survival risk scores **(A–C)**, survival status **(D–F)**, heatmaps of lncRNA expression **(G–I)**, Kaplan–Meier survival curves of OS **(J–L)** between risk groups in the train, validation, and entire sets, respectively.

Cuproptosis-related lncRNAs of the risk model were identified when the first-rank of Log(λ) in LASSO analysis achieved the minimum deviance (Figures 3A,B). Finally, we obtained 6 lncRNAs from the multi-Cox regression. These cuprotosis-related lncRNAs can be considered prognostic factors for CC. The correlation of lncRNAs with cuproptosis-related genes was analyzed, from which AJ003147.1 has the highest positive correlation with *DLST* (Figure 3C). Risk score of each individual was calculated as follows: risk score = AC096992.2 × (−0.5807) + AJ003147.1 × (−0.5740) + SOX21-AS1 × (−0.4562) + AL049869.2 × (2.5219) + CNNM3-DT × (−0.9408) + ARHGAP31-AS1 × (−0.9327). Figures 4A–C illustrate the risk score distribution. Compared to the individuals of low risk, the high-risk individuals had an increased death rate (Figures 4D–F). The heatmap revealed that AJ003147.1, AC096992.2, SOX21-AS1, CNNM3-DT, and ARHGAP31-AS1 had lower levels in individuals of the high risk compared to the low, whereas AL049869.2 was upregulated (Figures 4G–I). According to Kaplan-Meier analysis, patients with low risk outlived those with high risk (Figures 4J–L). The PFS of the CESC cohort was shown in Supplementary Figure S1.

### Independent analysis of the risk score

**FIGURE 5:**
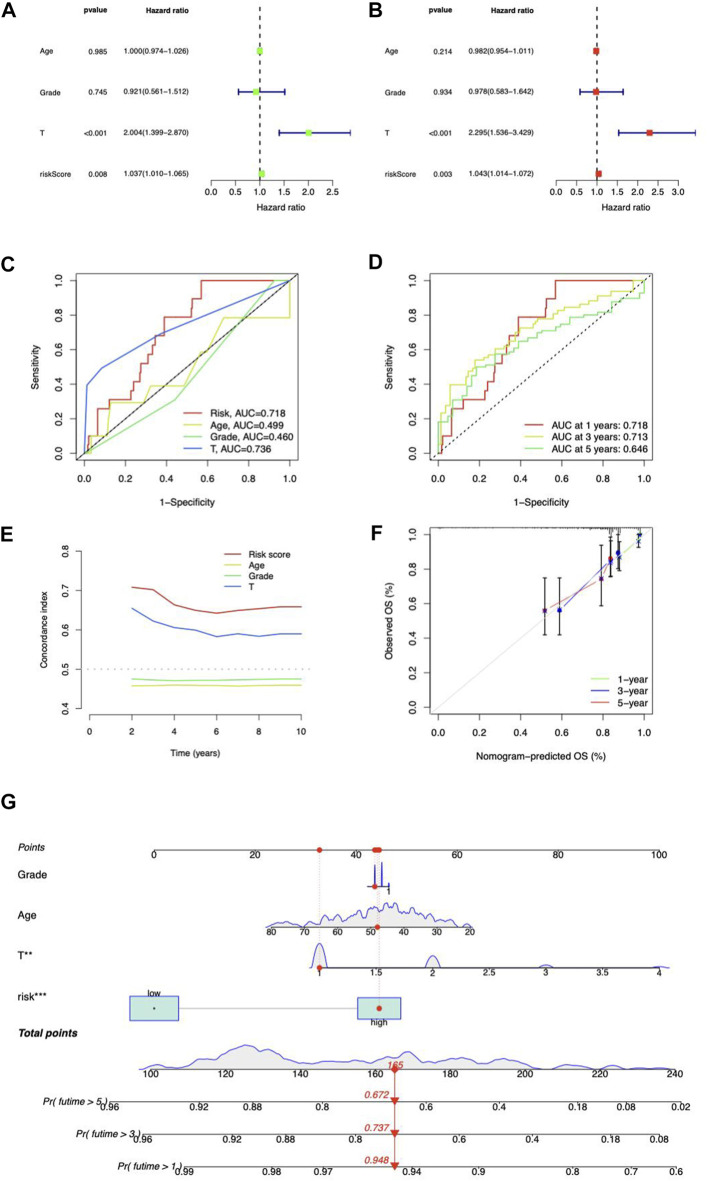
Prognosis value of risk model. **(A)** Univariate and **(B)** multivariate Cox regression of the independent analysis for prognosis. **(C)** Predictive accuracy of the risk model compared with age, Grade, and T stage. **(D)** Time-dependent ROC curve prediction. **(E)** C-index curve analysis. **(F)** Calibration curves in predicting outcomes at 1, 3, and 5 years. **(G)** Nomogram of risk score and clinicopathological variables and predicts 1-, 3-, and 5-year OS in CC.

Independent prognostic analysis was performed. The risk score and T stage were found related to OS analyzed by univariate and multivariate Cox regression (Figures 5A,B). Compared to other clinicopathological variables, the risk score’s area under the curve (AUC) showed a stronger predictive value (Figure 5C). For 1-year, 3-year, and 5-year survival, respectively, the AUC of the risk score was 0.718, 0.713, and 0.646 (Figure 5D). The risk score’s C-index score was likewise greater than the other clinical characteristics (Figure 5E). Above all, the risk score may function as an independent factor for CC prediction.

### Construction of the lncRNA-based nomogram

We coupled the risk score with clinicopathological variables to build a hybrid nomogram for 1-, 3-, and 5-year OS prediction in CC patients (Figure 5G). The calibration curves showed significant fitting between forecasts and measurements (Figure 5F).

### Relationships between risk scores and clinical features

**FIGURE 6:**
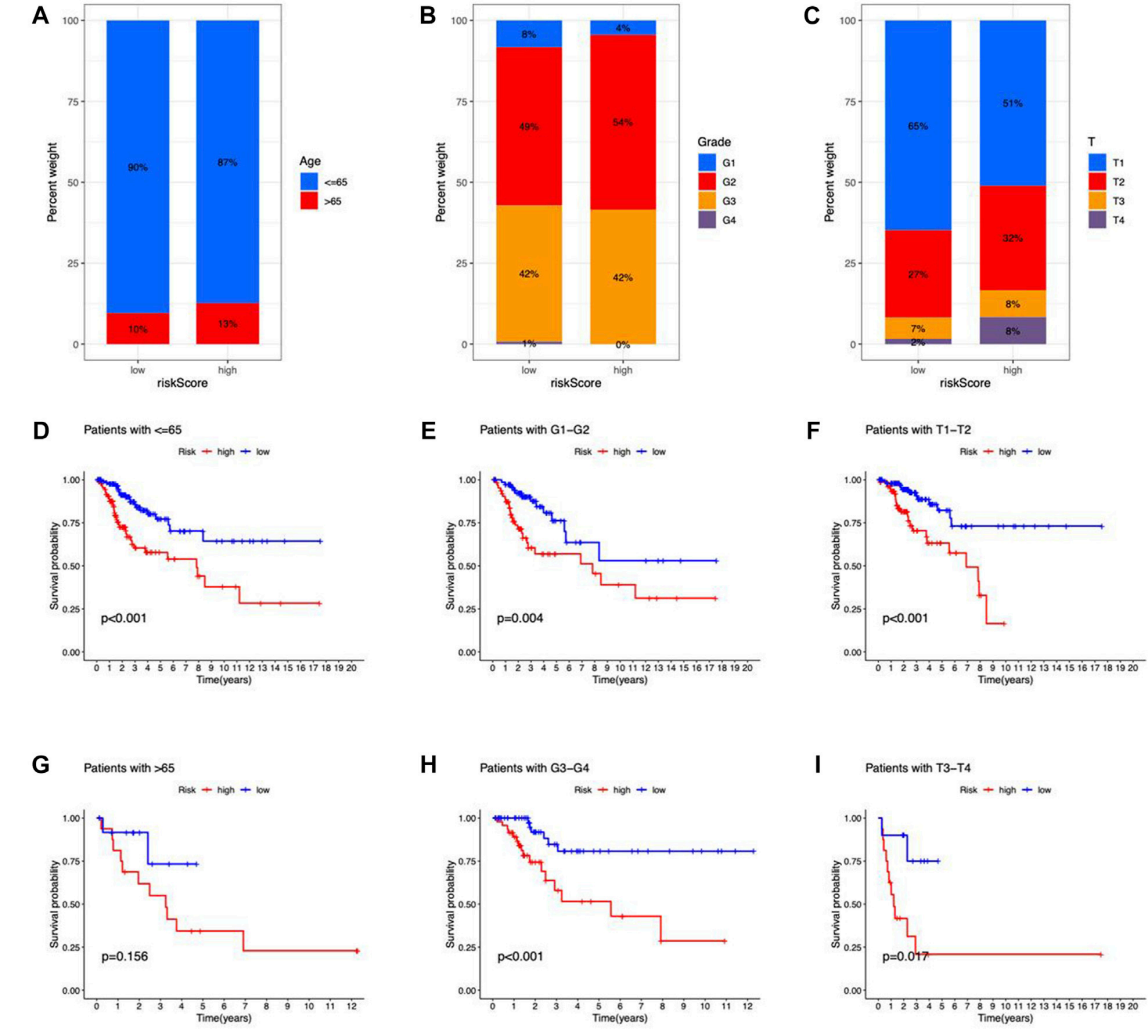
Clinicopathological features in the risk model. **(A–C)** Correlation between clinical characteristics and risk scores **(D–I)** Kaplan–Meier survival curves for subgroups analysis.

CC patients were grouped by age, Grade, and T stage to investigate the correlation between risk scores and clinical features. According to the proportion demonstrated in the columns, patients with age > 65, G2–G4, and T2–T4 may have higher risk scores (Figures 6A–C). Besides, the OS between risk groups in generic clinicopathological categories differed. CC patients were categorized into subgroups based on age (≤ 65 or > 65 years), Grade (G1–G2 or G3–G4), and T stage (T1–2 or T3–4). As shown in Figures 6D–I, patients of high risk had worse prognoses except for the age group of > 65. One potential explanation for the subgroup is the small number of patients due to the poor prognosis of advanced CC. According to the findings, the prognostic model could also be utilized for CC patients with distinct clinicopathological characteristics.

### Immune features of the risk model

**FIGURE 7:**
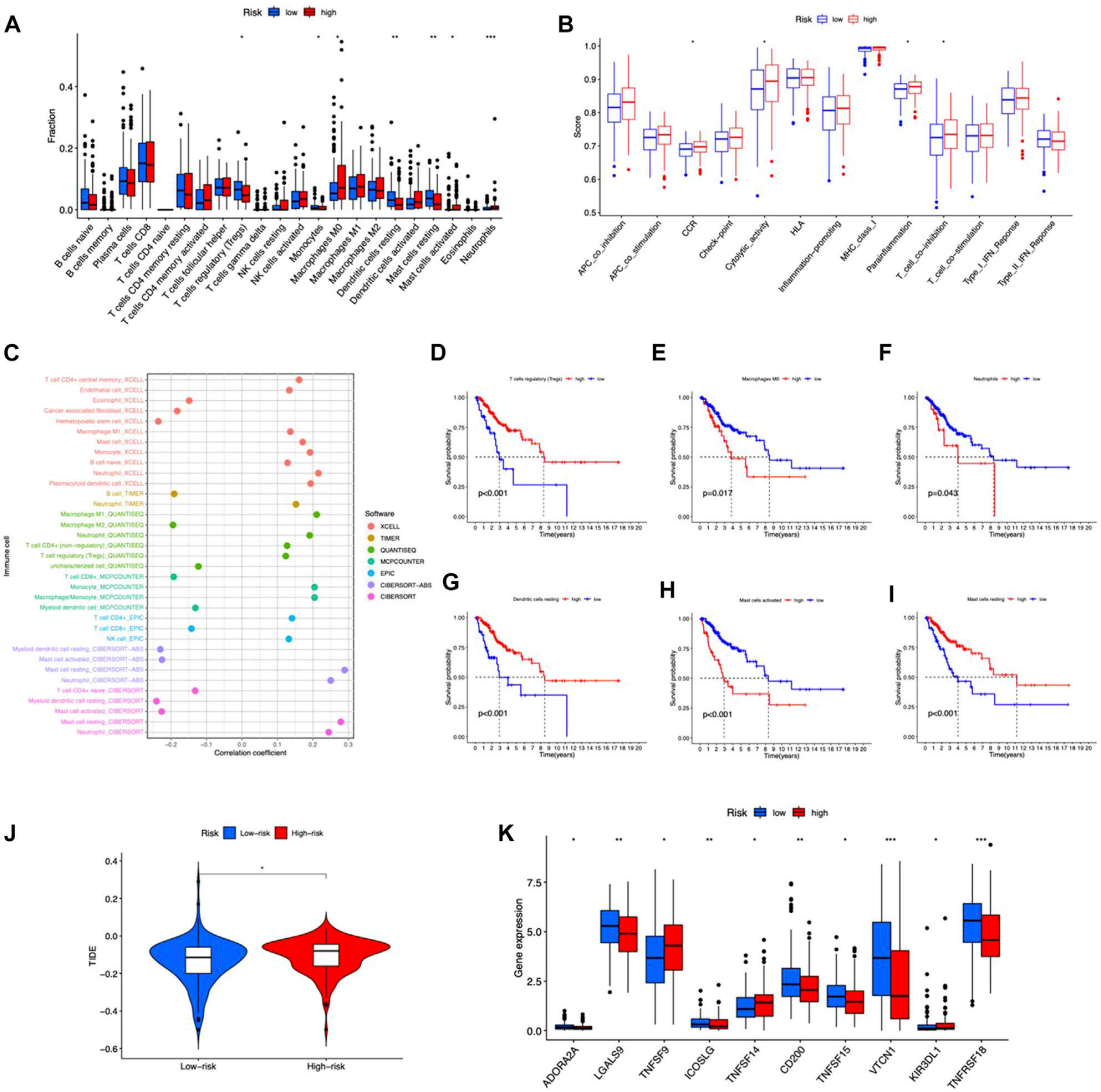
Tumor immune microenvironment in the risk model. **(A)** Different tumor-infiltrating lymph cells in patients with CC of high- and low-risk groups. **(B)** Immune functions of high- and low-risk groups. **(C)** Correlation between infiltrating immune cells and risk scores. **(D–I)** Kaplan–Meier survival curves for high- and low-level of different immunocytes. **(J)** TIDE scores between the two groups. **(K)** Differences in expression of immune checkpoints in the risk groups. *p < 0.05, **p < 0.01, ***p < 0.001.

We examined the correlation between risk score and immunocytes using the CIBERSORT method. The results showed a substantial difference in the proportion of infiltrating immunocytes between the two risk groups (Figure 7A). The immune functions between high- and low-risk groups were analyzed by ssGSEA, and the functions of CCR, cytolytic activity, parainflammation, and T cell co-inhibition were different in the two risk groups (Figure 7B). As a result, the proportion of resting mast cells, Tregs, monocytes, and resting dendritic cells exhibited higher levels in the low-risk group, while M0 macrophage, activated mast cells, and neutrophils had elevated levels in the high-risk group. Immune cells were shown to be strongly related to risk scores across multiple platforms. The correlation matrices of risk score and tumor-infiltrating immune cells are shown in Figure 7C. From the bubble graphs, the risk scores were positively correlated with infiltration of Mast cell resting, Neutrophil, Monocyte, Macrophage M1, and plasmacytoid dendritic cell and negatively correlated with infiltration of Mast cell activated, Myeloid dendritic cell resting, and Hematopoietic stem cell. Besides, we compared the OS of immunocytes of high- and low-proportion categories and found high Macrophages M0, Neutrophils, and Mast cells activated and low Tregs, Dendritic cells resting and Mast cells resting were associated with poorer OS (Figures 7D–I). Furthermore, TIDE score prediction showed that observations of high risk exhibited higher TIDE scores compared to the low risk, indicating a higher antitumor immune escape possibility of the high-risk group (Figure 7J). The immune checkpoint gene expression analysis showed higher expression levels in observations of low risk (Figure 7K), implying that individuals of low risk may benefit more from immune checkpoint inhibitor (ICI) treatment than the high risk. Taken together, the immunological microenvironment varied across risk groups, which might partly explain the considerable disparity in prognosis between them.

### Analyses of enriched pathways between risk groups

**FIGURE 8:**
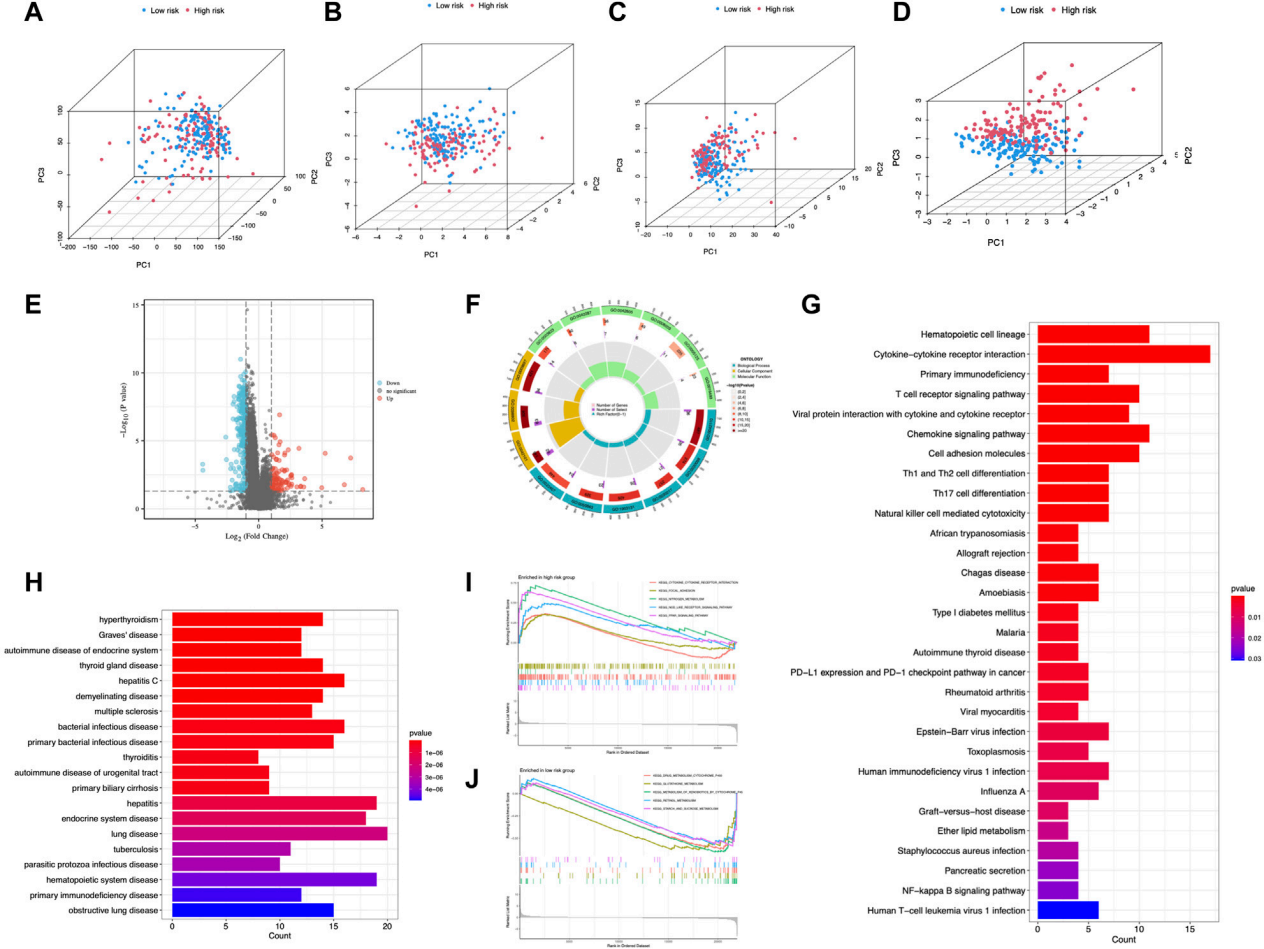
PCA and enrichment analysis in different risk groups. PCA analysis of risk groups based on **(A)** the expression of all examined genes, **(B)** cuproptosis-associated genes, **(C)** cuproptosis-associated lncRNAs, and **(D)** the 6 lncRNAs of the risk model. **(E)** DEGs between risk groups. GO **(F)**, KEGG **(G)**, and DO **(H)** enrichment of DEGs. GSEA of the high-risk group **(I)** and low-risk group **(J)**.

Principal components analysis (PCA) suggested that CC cases could be classified into two distinct clusters according to expression profiles of the total genes, cuproptosis genes, cuproptosis-related lncRNAs, and risk lncRNAs (Figures 8A–D), exhibiting a good discriminating power. Furthermore, 266 DEGs between different risk groups were identified (55 upregulated and 211 downregulated) (Figure 8E). GO analysis revealed that DEGs were mainly enriched in T cell activation, lymphocyte differentiation, and T cell receptor complex (Figure 8F; Supplementary Figure S2). DEGs were mainly enriched in cytokine-cytokine receptor interaction and T cell receptor signaling pathway from KEGG analysis (Figure 8G). DO enrichment analysis resulted mainly in hyperthyroidism, Graves’ disease, and autoimmune disease of the endocrine system (Figure 8H). GSEA demonstrated the gene profile of the high-risk group was mainly associated with cytokine-cytokine receptor interaction, nitrogen metabolism, NOD-like receptor signaling pathway, and PPARγ signaling pathway (Figure 8I). Metabolism pathways were enriched in the low-risk group (Figure 8J). These results demonstrated the involvement of immune-related and metabolic biological processes and pathways in the development of CC.

### Chemotherapeutics prediction

**FIGURE 9:**
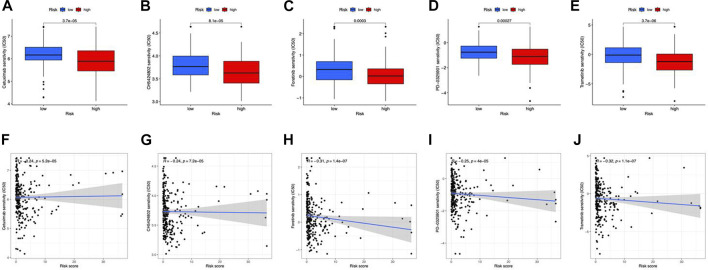
Drug susceptibility prediction. **(A–E)** Sensitive drugs in high-risk groups. **(F–J)** Correlation between sensitive drugs and risk scores.

In terms of therapeutic application potential, we studied the drug sensitivity of the two risk categories. Totally, 5 compounds showed substantial sensitivity in the high-risk category (Figures 9A–E). Among them, cetuximab is used as the first-line medication for colorectal cancer patients (Qin et al., 2018). CH5424802, also known as alectinib, is a selective, and orally available ALK inhibitor with preferential antitumor activity (Sakamoto et al., 2011). In the main therapy of ALK-positive NSCLC, alectinib outperformed crizotinib in terms of effectiveness and toxicity (Peters et al., 2017). In advanced hepatocellular carcinoma patients, foretinib displayed promising antitumor efficacy and tolerability in the first-line therapy (Yau et al., 2017). PD-0325901 (a MAPK/ERK kinase inhibitor) (Solares et al., 2021), and Trametinib (an oral MEK inhibitor) (Planchard et al., 2017) were also applied in clinical. Besides, we found that all the sensitive drugs were correlated with risk scores (Figures 9F–J).

## Discussion

Cervical cancer (CC) is a major public health issue in low and middle-income nations due to a lack of comprehensive screening programs (Tsikouras et al., 2016). Squamous cell carcinomas and adenocarcinomas are the most common histological kinds of CC, accounting for around 70% and 25% of all cervical malignancies, respectively (He et al., 2020; Park, 2020). HPV is the most prevalent but not the only risk factor for cervical cancer (He et al., 2020). Over 70% of HPV infections are caused by high-risk HPV, such as serotypes 16 and 18 (Small et al., 2017). It is challenging to access novel medicines for CC, in part due to immunological resistance. This necessitates the discovery of new biomarkers for monitoring CC development as well as therapeutic targets to increase survival rates.

Cuproptosis, a newly found kind of unique cell death, may reenergize research into the use of copper to treat cancer (Kahlson and Dixon, 2022). Copper is indispensable for sustained life activities since it is involved in mitochondrial respiration and iron absorption (Wang et al., 2022). However, it may be hazardous with the copper overloaded. There is currently little understanding of how copper regulates mortality in cancers. Copper ionophore treatment is more effective for cancers that express large levels of lipoylated tricarboxylic acid enzymes and undertake mitochondrial respiration (Tsvetkov et al., 2022). Despite the lack of clear evidence regarding the role of copper death-associated lncRNAs in CC, using copper toxicity may provide a promising therapeutic approach.

In CC pathology, lncRNAs appear to play a vital role in sustaining multiple biological activities. lncRNA abnormalities are linked to the onset and progression of CC (He et al., 2020). However, studies on cuproptosis-related lncRNAs that predict CC survival are scarce. Here, we sorted out 6 prognostic lncRNAs associated with cuproptosis-related genes (AJ003147.1, AC096992.2, SOX21-AS1, AL049869.2, CNNM3-DT, and ARHGAP31-AS1). SOX21-AS1, a multifunctional molecule involved in the immune- (Xu et al., 2022b), m7G- (Shao et al., 2022), and m6A-related (Xie et al., 2022) biological process in tumors, may impact CC etiology and prognosis. Li et al. demonstrated that SOX21-AS1 promotes cell proliferation, migration, and invasion in breast cancer (Li et al., 2021). The SOX21-AS1/miR-144-3p/PAK7 axis was shown to be carcinogenic in glioma cells through the Wnt/β-catenin pathway (Gai and Yuan, 2020) and SOX21-AS1 was found upregulated in lung cancer (Wang et al., 2021). In CC, SOX21-AS1 showed hypomethylation (Du et al., 2021) and could promote CC progression (Zhang et al., 2019). In addition, the heatmap of gene-lncRNA expression correlation (Figure 3C) indicates that lncRNA AJ003147.1 has a highly positive correlation with *DLST*. *DLST* is a glutamine regulator found in the tricarboxylic acid cycle (TCA) for oxidative decarboxylation. In individuals with neuroblastoma, a high *DLST* predicts poor treatment outcomes (Anderson et al., 2021). Suppression of the TCA cycle through *DLST* depletion decreases the invasion ability of triple-negative breast cancer (TNBC) (Shen et al., 2021). Targeting glutamine metabolism in immunosuppressive myeloid cells with *DLST* reduced their activity, resulting in a reduction in the immunosuppressive tumor microenvironment and therefore improving the effectiveness of immunotherapy in ovarian cancer (Udumula et al., 2021). LncRNAs are small RNAs precursors (Cheetham et al., 2013) and stimulate mRNA alternative splicing (Romero-Barrios et al., 2018). They affect protein transport and localization *via* post-translationally binding to targets (Huang and Zhang, 2014). In the present study, the selected lncRNAs all positively correlated with cuprotosis genes, indicating a potential regulatory function and cuproptosis targets for CC treatment. The remaining lncRNAs were demonstrated for the first time. Newly discovered knowledge of cuproptosis-related lncRNAs might help bring a breakthrough into clinical practice by improving the mechanistic understanding of CC.

We constructed a predictive risk model using 6 cuproptosis-related lncRNAs and our suggested model’s AUCs and C-index scores were higher than the other clinical features of CC patients, showing remarkable prediction potential. Furthermore, the risk score was substantially related to the clinical characteristics of CC. The risk model showed capability for independent prognostic prediction, and the survival curve findings demonstrate that the model is clinically applicable to CC patients.

For the treatment of CC, it offers great promise to combine immunogenic therapeutics with novel immunotherapeutic regimens (Attademo et al., 2020; Ferrall et al., 2021). The two risk groups exhibited distinct immunological microenvironments. Furthermore, high-risk individuals had increased activated Mast cells and decreased resting mast cells (Figure 7A) and showed a poorer activated Mast cells-related OS, as well as a better resting mast cells-related OS (Figures 7G,H), suggesting that the subgroup- or stated-related role of mast cells in TME. Generally, activated immune cells release proinflammatory cytokines and chemokines, which promote tumor cell growth (Ferrari et al., 2019). Mast cells release matrix metalloproteinases (MMPs) that aid tumor invasion and they actively stimulate neovascularization by producing conventional proangiogenic factors like VEGF, PDGF, and IL-6 (Komi and Redegeld, 2020). To further evaluate the practical applicability of immunotherapy, we assessed the TIDE score of the two groups. Observations of low-risk had lower TIDE scores, which suggests that, according to our risk model, low-risk patients would acquire more benefits from immunotherapy. Moreover, in line with the results of immune checkpoint detection, the low-risk group also showed a more robust checkpoint gene expression. Based on these findings, our signature might be a possible measure to evaluate the efficacy of immunotherapy in CC patients, and immune checkpoint inhibitor (ICI) medication may be a good option for low-risk patients. Aside from immunotherapy, we discovered chemotherapy prediction based on the risk model, and individuals of high risk were quite sensitive to the clinically applied therapy medications. These findings show that high-risk patients react better to chemotherapy and targeted medicines, which is significant for personalized cancer treatment. Overall, these discoveries might lead to new therapeutic options for CC sufferers and affect individualized tumor therapy.

Identifying various risk stratification groups’ signaling pathways may aid in a better understanding of the molecular biology process in CC. We observed that the DEGs of the two risk categories were associated with immune-related and metastatic processes utilizing the GO, KEGG, and DO analyses. GSEA showed DEGs enriched in the KEGG cytokine-cytokine pathway, which influences autoimmune, inflammatory, and viral disorders (Spangler et al., 2015). Our results suggested immune-related processes affected CC development, which was mediated by cuproptosis-related lncRNAs. This work offers a new foundation for future therapeutic methods by elucidating the involvement of cuproptosis-related lncRNAs in CC immune modulation.

The study’s key findings and implications were as follows. To begin, this work involves the first development and comprehensive investigation of a unique 6-cuproptosis-related lncRNA risk model to predict CC patients’ prognosis. Second, risk score in the model was found connected to clinicopathological characteristics, immune infiltration, and TIDE score change, which may shed light on tumor state prediction and antitumor response, potentially giving targets for future therapeutic intervention. Third, sensitive drug prediction might be a possible therapeutic technique for improving the effectiveness of CC immunotherapy, and providing tailored ramifications for personalized cuproptosis-related lncRNAs may predict prognosis and aid in the development of a viable treatment approach in CC. This would significantly advance individual therapy and enhance patient outcomes. Cuproptosis-related lncRNA targeting is a potential strategy to overcome systemic treatment failures and broaden immunotherapy. These findings might help individuals with CC who are undergoing immunotherapy or chemotherapy treatment. Despite multiple aspects of our verification, the risk model still has some limitations. First, it is a retrospective analysis and there are evitable selection and recall biases. Second, this study lacks *in vivo* and *in vitro* experiments to validate the functional roles of the lncRNAs. Third, the model was solely evaluated internally by TCGA, considering genetic heterogeneity, these lncRNAs need to be validated in more public databases, and also a larger sample size is required to obtain conclusive findings, and the prediction model should be practically verified before being applied to clinical patients.

## Conclusion

In conclusion, we developed a unique 6 cuproptosis-related lncRNA risk model that might be used as a predictive technique for CC patients. The immune checkpoint inhibitors and chemosensitivity were evaluated, as well as the association between the risk model and the immunological environment. These observations may benefit immune therapy-based interventions and chemotherapy in patients with CC.

## Data Availability

The original contributions presented in the study are included in the article/Supplementary Material, further inquiries can be directed to the corresponding author.

## Supplementary material

Supplementary Figure S1 PFS of the CESC cohort.

Supplementary Figure S2 DEGs of high- and low-risk groups.

Supplementary Figure S3 GO enrichment analysis.
