# Supplementary material for: Comprehensive analysis of cuproptosis-related lncRNAs model in tumor immune microenvironment and prognostic value of cervical cancer
Source: Front Pharmacol. 2022 Nov 30;13:1065701. doi: 10.3389/fphar.2022.1065701 (PMC9747936; doi:10.3389/fphar.2022.1065701)
Supplement: Supplementary file 1 [file Table1.DOCX]

| Supplementary Table 1. Clinical information of patients in high- and low-risk groups. | | | | |
| --- | --- | --- | --- | --- |
| Features | Total | Test | Train | *p* value |
| Age |  |  |  | 0.5906 |
| <=65 | 242(88.97%) | 122(90.37%) | 120(87.59%) |  |
| >65 | 30(11.03%) | 13(9.63%) | 17(12.41%) |  |
| Grade |  |  |  | 0.784 |
| G1 | 16(5.88%) | 8(5.93%) | 8(5.84%) |  |
| G2 | 126(46.32%) | 63(46.67%) | 63(45.99%) |  |
| G3 | 103(37.87%) | 53(39.26%) | 50(36.5%) |  |
| G4 | 1(0.37%) | 0(0%) | 1(0.73%) |  |
| unknow | 26(9.56%) | 11(8.15%) | 15(10.95%) |  |
| T |  |  |  | 0.971 |
| T1 | 128(47.06%) | 64(47.41%) | 64(46.72%) |  |
| T2 | 64(23.53%) | 33(24.44%) | 31(22.63%) |  |
| T3 | 16(5.88%) | 9(6.67%) | 7(5.11%) |  |
| T4 | 10(3.68%) | 5(3.7%) | 5(3.65%) |  |
| unknow | 54(19.85%) | 24(17.78%) | 30(21.9%) |  |
