# Supplementary figures and images for: Comprehensive analysis of cuproptosis-related lncRNAs model in tumor immune microenvironment and prognostic value of cervical cancer
Source: Front Pharmacol. 2022 Nov 30;13:1065701. doi: 10.3389/fphar.2022.1065701 (PMC9747936; doi:10.3389/fphar.2022.1065701)

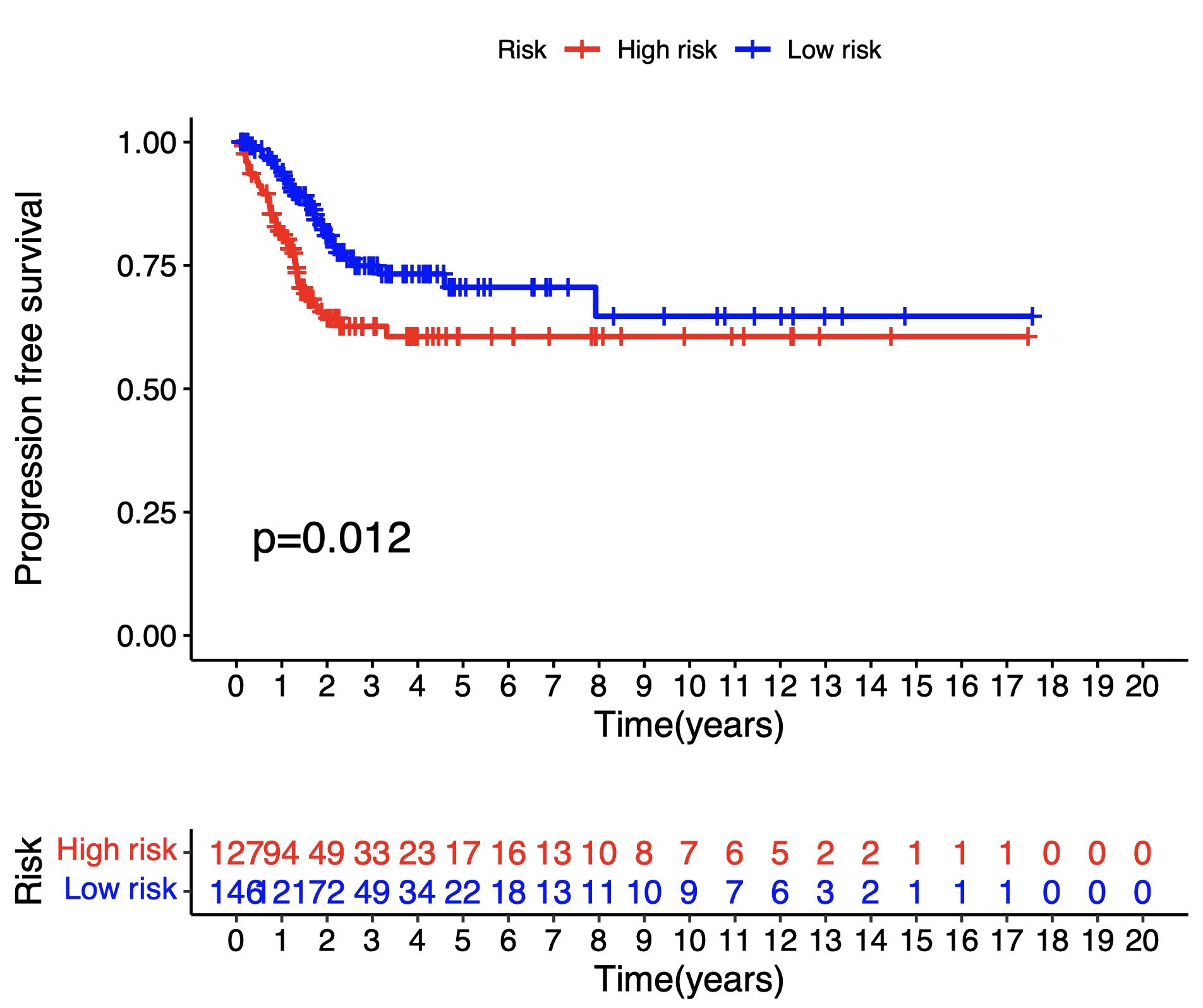

Supplement: Supplementary file 2 [file Image1.JPEG]

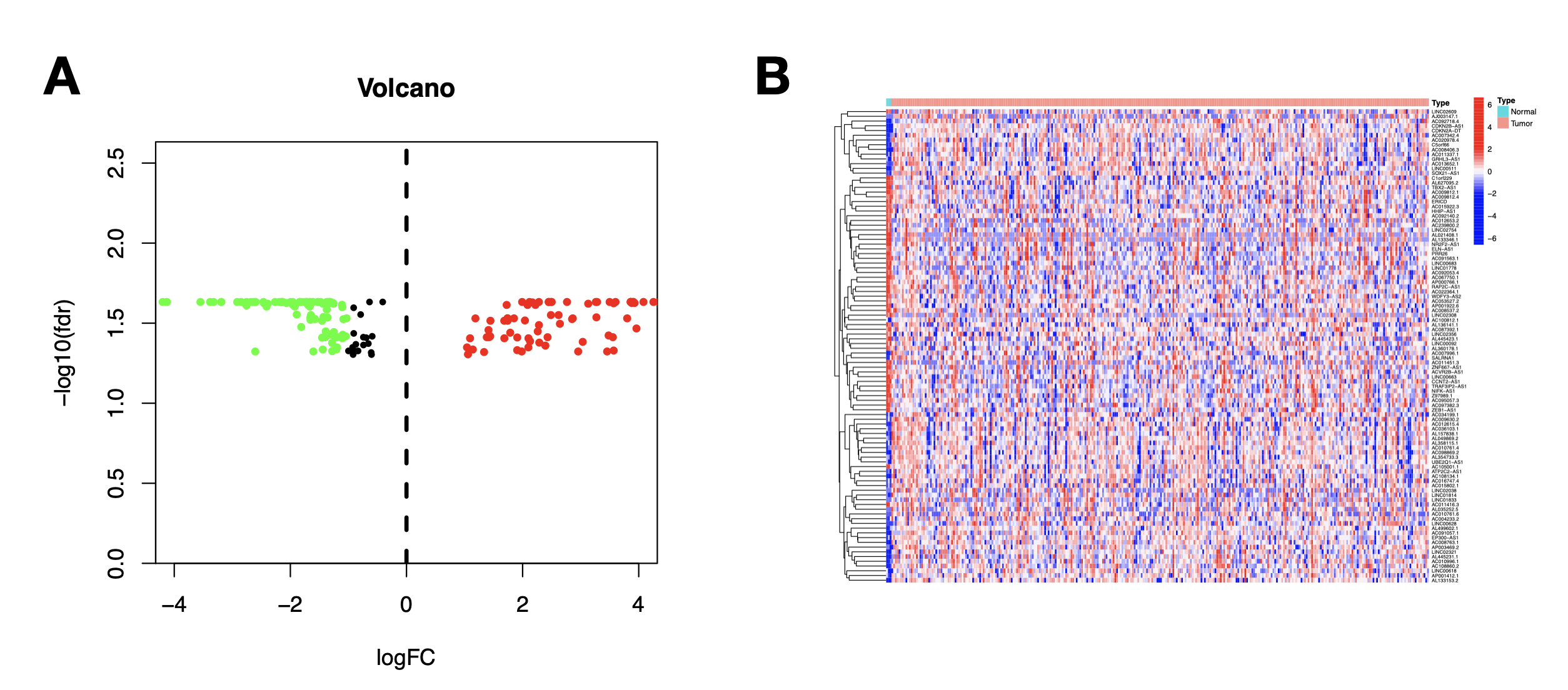

Supplement: Supplementary file 3 [file Image2.PNG]

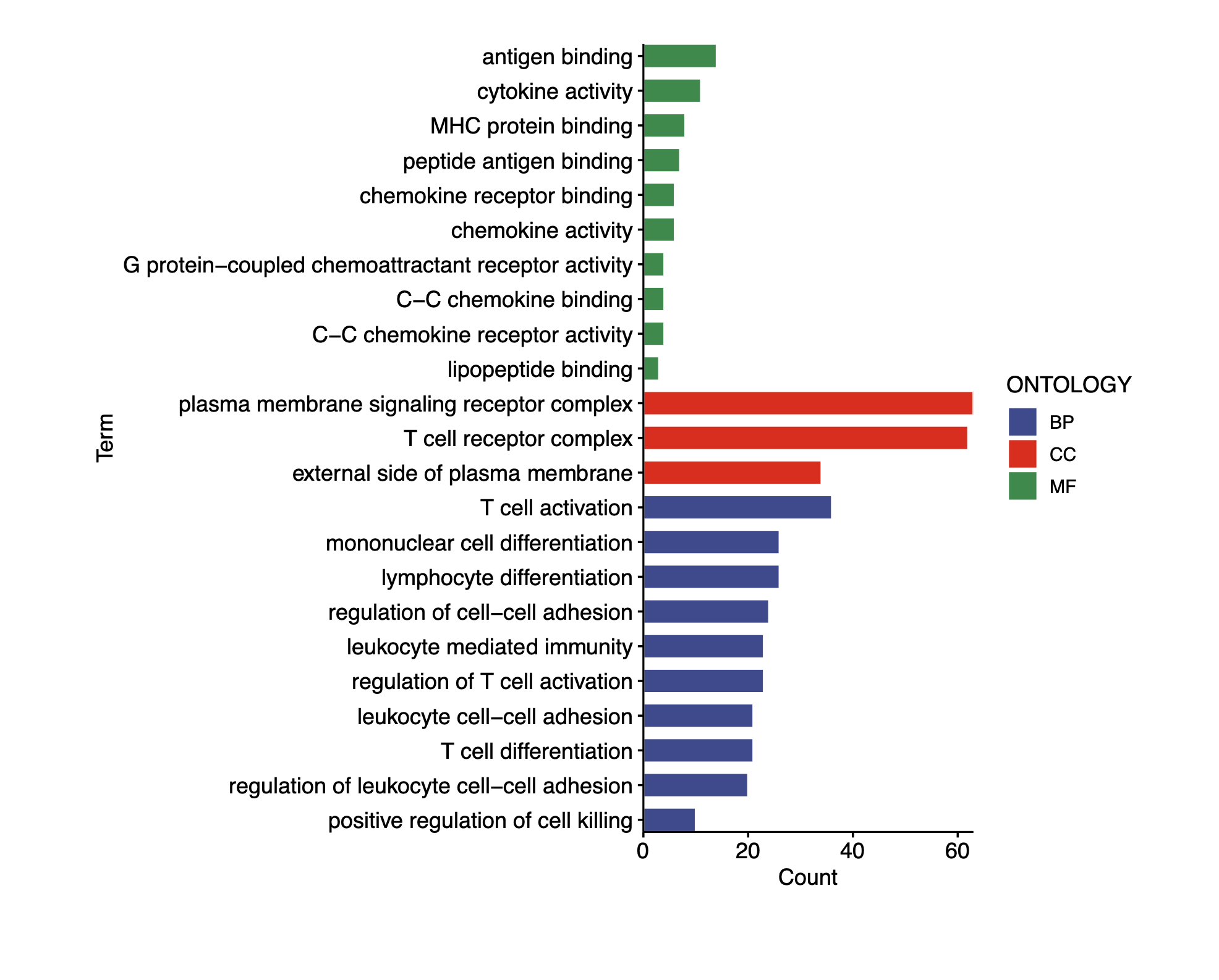

Supplement: Supplementary file 4 [file Image3.PNG]
